# Foliar Application of dsRNA Suppresses Watermelon Bud Necrosis Virus Infection and Symptom Development in *Nicotiana benthamiana* and Cowpea Plants

**DOI:** 10.3390/plants15203237

**Published:** 2026-10-21

**Authors:** Dipinte Gupta, Prabu Gnanasekaran, Oinam Washington Singh, Suryakant Manik, Anirban Roy, Bikash Mandal

**Affiliations:** 1 Advanced Centre for Plant Virology, Division of Plant Pathology, Indian Agricultural Research Institute, New Delhi 110012, India; dipinte.gupta@wsu.edu (D.G.); oinamw@gmail.com (O.W.S.); suryamanik94@gmail.com (S.M.); 2 Department of Plant Pathology, Washington State University, Pullman, WA 99164, USA; prabu.gnanasekaran@wsu.edu; 3 Indian Council of Agriculture Research, Central Institute for Cotton Research, Regional Station, Sirsa 125055, Haryana, India; 4 Indian Council of Agriculture Research, Indian Institute of Agricultural Biotechnology, Ranchi 834003, Jharkhand, India

**Keywords:** WBNV, RNAi, dsRNA, mesoporous silica nanoparticle

## Abstract

Watermelon production is significantly affected by watermelon bud necrosis virus (WBNV), an economically important orthotospovirus in the Indian subcontinent. RNA interference (RNAi)-based approaches offer promising strategies for developing broad-spectrum antiviral resistance in susceptible plants. In this study, we evaluated the efficacy of foliar application of double-stranded RNA (dsRNA) and hairpin RNA (hpRNA) derived from the full-length *NSs* gene of WBNV against WBNV infection in two host plants, *Nicotiana benthamiana* and cowpea (*Vigna unguiculata*). Fluorescein-labeled dsRNA of *NSs* gene (dsNSs) was tracked in *N. benthamiana* plants, where dsNSs translocated from the treated leaf to adjacent leaves, stem, and roots. Naked dsNSs was no longer detectable in treated leaves at 5 days post-inoculation (dpi), whereas mesoporous silica nanoparticle-conjugated dsNSs (dsNSs-MSNP) remained detectable in the treated leaves beyond 5 dpi, indicating enhanced stability. Following WBNV inoculation, *N. benthamiana* plants treated with hairpin of *NSs* gene (hpNSs), dsNSs, and dsNSs-MSNP exhibited 112, 303, and 370 fold reduction in viral RNA accumulation, respectively. Although these treatments delayed symptom development, all inoculated plants eventually showed the viral symptoms in *N. benthamiana*. Similarly, treatment of *Vigna unguiculata* cv. Pusa Komal (cowpea) plants with hpNSs, dsNSs, and dsNSs-MSNP resulted in reduced viral load, where dsNSs-MSNP provided the maximum reduction up to 208 fold. The dsNSs provided 100% reduction in development of systemic symptoms of bud necrosis disease in the treated cowpea plants. Collectively, these findings demonstrate that foliar application of nanoparticle-conjugated dsRNA targeting the full-length *NSs* gene can effectively suppress WBNV infection and provide enhanced protection against disease development in cowpea.

## 1. Introduction

Watermelon (*Citrullus lanatus*) is an economically important crop cultivated extensively in India [1]. However, watermelon production is severely constrained by a bud necrosis disease caused by watermelon bud necrosis virus (WBNV; *Orthotospovirus citrullonecrosis*), a member of the genus Orthotospovirus [2]. WBNV virion is quasi-spherical and contains a tripartite RNA genome comprising of L, M, and S segments. The L RNA encodes the viral RNA-dependent RNA polymerase. The M segment encodes NSm and a glycoprotein precursor, while the S segment encodes the nonstructural protein NSs and the nucleocapsid protein N, which encapsidates the viral RNAs [3,4,5]. The abundance of both WBNV and the vector thrips and lack of resistance in available watermelon cultivars pose a major challenge for the effective management of bud necrosis disease [6]. Among the various approaches explored for managing viral diseases in plants, RNA interference (RNAi) has emerged as a promising strategy for inducing antiviral resistance in plant against a specific virus. By targeting essential viral genes, RNAi can suppress viral replication and movement and, consequently, reduce disease development [7,8].

RNAi is an important component of plant growth, development, and innate defense against invasive nucleic acids, including transposable elements, viruses, and virus-like pathogens [9,10]. In antiviral defense, plants employ post-transcriptional gene silencing (PTGS), whereby viral RNA is specifically recognized and degraded, thereby limiting virus accumulation and spread [11,12]. During this process, viral dsRNA or partially overlapping viral transcripts are processed by Dicer-like (DCL) enzymes into small interfering RNAs (siRNAs), typically 21–24 nucleotides in length [13]. These siRNAs are subsequently loaded onto Argonaute (AGO) proteins to form the RNA-induced silencing complex (RISC), which recognizes and degrades complementary viral RNA transcripts [14]. In addition, RNA-dependent RNA polymerases (RDRs) can use complementary RNA molecules as templates to generate secondary siRNAs, thereby amplifying and reinforcing the silencing response. Exploitation of this endogenous antiviral mechanism has therefore become an important strategy for developing crops with enhanced resistance to viruses and other pathogens [15].

The RNAi pathway has been extensively exploited to engineer virus resistance by genetic modification (GM) of plan through the introduction of a portion of viral sequence into plant genome. Stable transformation approaches have successfully generated virus-resistant transgenic cultivars in several economically important crops [16,17]. However, despite the effectiveness, transgenic approach is associated with several limitations, including lengthy and labor-intensive transformation procedures, substantial resource requirements, and strict government regulation policy. These limitations have stimulated the interest in non-transgenic approach for induction of antiviral RNAi in plants through the external application of virus-derived dsRNA. The topical or exogenous application of dsRNA has emerged as a promising non-GMO strategy for plant virus management as it can induce sequence-specific antiviral responses without requiring the stable integration of foreign sequences into the plant genome [18]. In contrast to the transgenic approach, exogenous application of dsRNA provides a relatively a rapid, inexpensive and flexible approach for evaluating RNAi against viral infection.

The efficacy of exogenously applied dsRNA has been demonstrated against several plant viruses [18,19,20]. The studies involving other important tospoviruses such as tomato spotted wilt virus (TSWV) and groundnut bud necrosis virus (GBNV) have demonstrated the potentiality of exogenous dsRNA for suppressing tospovirus infection [21,22,23,24]. Furthermore, the effectiveness of topical dsRNA can be influenced by its stability, uptake, and movement within plant tissues. Thus, strategies capable of improving dsRNA stability and delivery may enhance the efficacy and persistence of RNAi-mediated antiviral protection. However, despite the economic importance of WBNV and the limitations associated with its management, the potential of exogenously applied dsRNA for controlling WBNV has not been investigated so far.

In the present study, we evaluated for the first time and compared the protective efficacy of three versions of dsRNA against WBNV infection: an NSs-derived hairpin RNA construct (hpNSs), dsRNA targeting the *NSs* gene (dsNSs) and mesoporous silica nanoparticle-conjugated dsNSs (dsNSs-MSNP). Their antiviral efficacy was assessed in *N. benthamiana* and *Vigna unguiculata* cv. Pusa Komal (cowpea). In addition, the translocation and persistence of dsNSs and dsNSs-MSNP were investigated to determine whether MSNP-mediated delivery could improve the stability and movement of exogenously applied dsRNA within plant tissues. Collectively, this study demonstrates the potential of exogenous RNA-based strategies for WBNV management and provides insights into the use of MSNPs as a delivery platform with enhanced dsRNA-mediated antiviral protection.

## 2. Results

### 2.1. Generation of dsRNA and Hairpin of NSs Gene of WBNV

**Figure 1:**
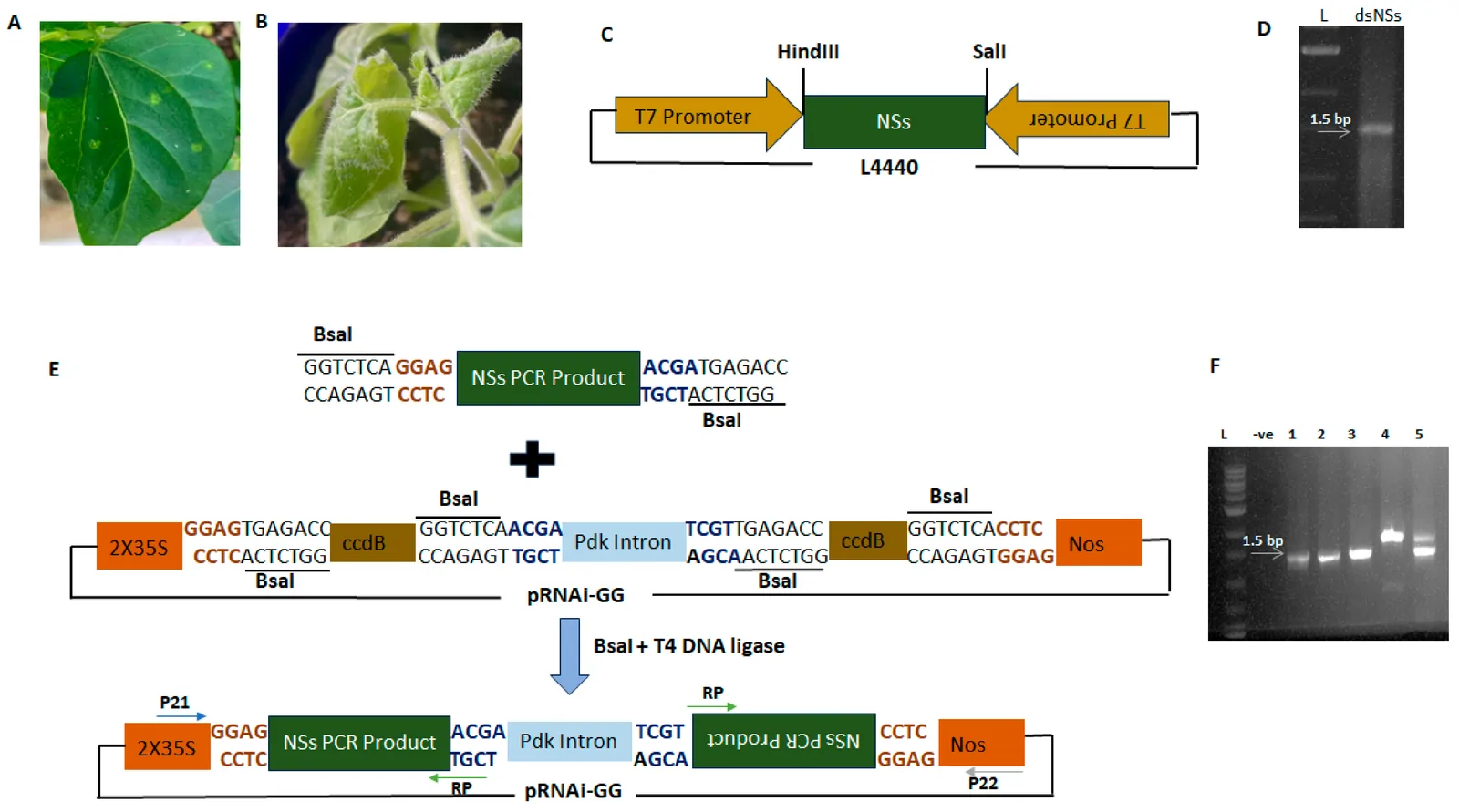
Establishment of watermelon bud necrosis virus (WBNV) infection and development of NSs-based RNAi constructs. (**A**,**B**) WBNV infection in *Nicotiana benthamiana* and cowpea using field-collected samples under plant growth chamber conditions. (**C**) Schematic representation of the cloning of the WBNV NSs gene into the L4440 vector for dsRNA production. (**D**) Purification of NSs-specific dsRNA (dsNSs) from transformed *Escherichia coli* HT115 cells. (**E**) Schematic representation of the development of the NSs hairpin construct (hpNSs). (**F**) PCR-based confirmation of the hpNSs. PCR products were resolved on a 1.2% agarose gel. -e: no-template control; Lane 1: PCR amplification of the NSs gene; Lane 2: PCR amplification of the NSs gene containing BsaI restriction sites; Lane 3: PCR amplification using P21 and the NSs reverse primer, confirming one arm of the inverted repeat; Lane 4: PCR amplification using P22 and the NSs reverse primer, confirming the second arm of the inverted repeat; Lane 5: PCR amplification using P21, P22, and the NSs reverse primer, confirming the presence of both inverted arms separated by the intronic sequence. L: DNA marker 1 kb Plus DNA Ladder (G-Biosciences, St. Louis, MO, USA).

The field isolate of WBNV was confirmed by PCR using WBNV *N* gene specific primers, which produced the expected amplicon of approximately 800 bp, confirming the identity of the virus isolate. The symptoms developed in *N. benthamiana* and cowpea following inoculation with the field-collected sample under growth chamber conditions are shown in Figure 1A,B, respectively. The *NSs* gene (1320 bp) of WBNV was successfully cloned into the L4440 vector and sequenced. dsRNA against the *NSs* gene of WBNV was produced and purified from *Escherichia. coli* strain HT115 (Figure 1C,D). In parallel, a hairpin construct targeting the WBNV *NSs* (hpNSs) gene was successfully cloned into the pRNAiGG. The presence of the NSs hairpin was confirmed by PCR amplification of the expected band (Figure 1E,F), confirming the successful generation of the hairpin construct.

### 2.2. Functionalized MSNP Conjugates with dsRNA and Translocate in Plant

**Figure 2:**
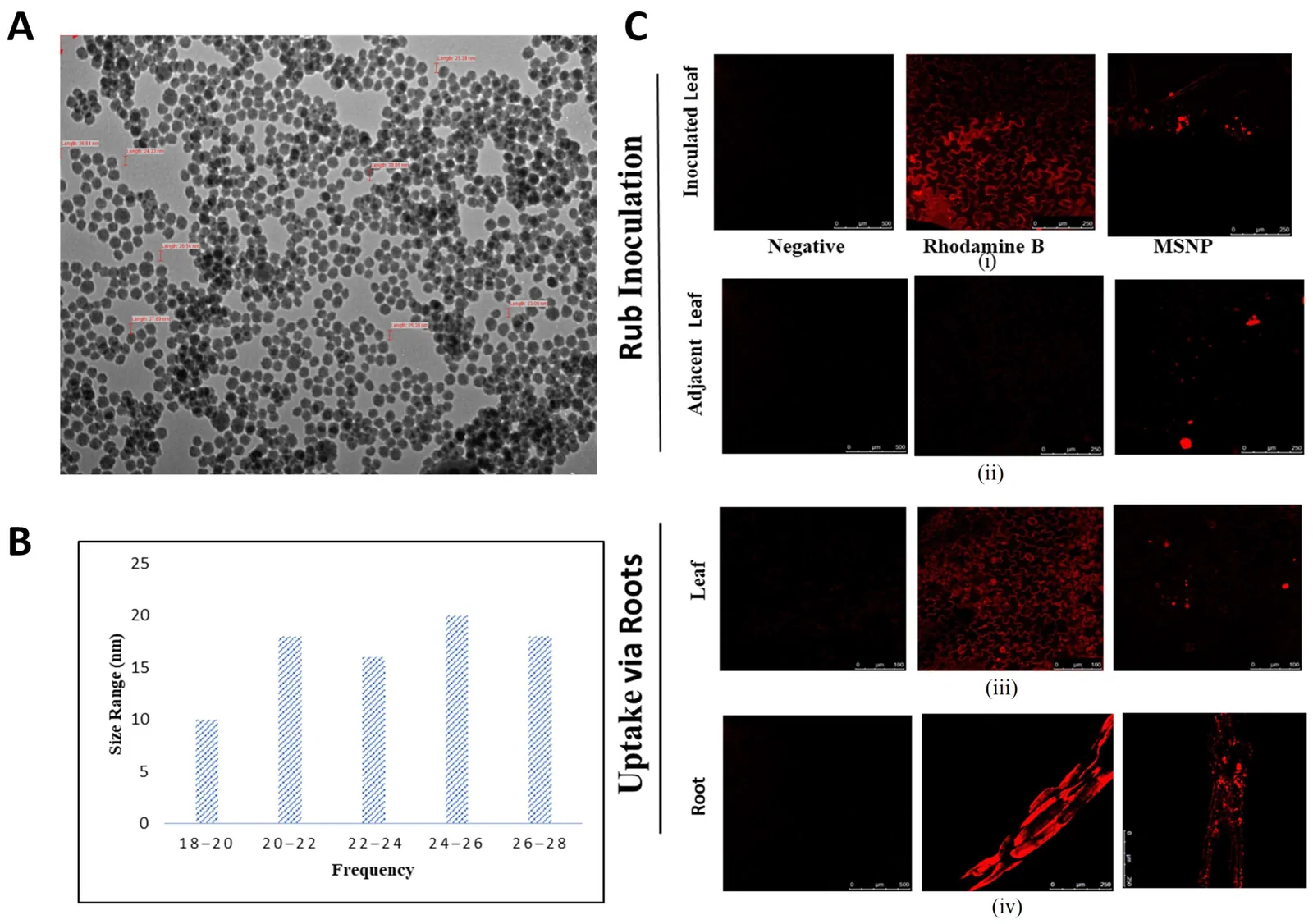
Synthesis, size, surface charge characterization, and translocation of mesoporous silica nanoparticles (MSNPs) in *Nicotiana benthamiana*. (**A**) Transmission electron microscopy image showing the synthesized MSNPs. (**B**) Particle size distribution of the synthesized MSNPs, showing a size range of approximately 18–28 nm. (**C**) Translocation of rhodamine-labeled MSNPs in *N. benthamiana*. Rhodamine-labeled MSNPs were detected in adjacent leaves (**i**,**ii**) following foliar application to a rub-inoculated leaf and were also detected in (**iii**,**iv**) leaves following root-mediated uptake.

The synthesized MSNPs were characterized by transmission electron microscopy, which revealed an average particle size of approximately 24 nm (Figure 2A,B). To facilitate electrostatic interaction with negatively charged dsRNA, the MSNP surfaces were functionalized with 3-aminopropyltriethoxysilane to introduce positively charged amino groups. Successful surface functionalization was confirmed by zeta potential analysis, with the average surface potential of the unfunctionalized MSNPs −30 mV and amine functionalized MSNPs +10 mV (Figure S1).

The negatively charged dsRNA was subsequently loaded onto the positively charged amino-functionalized MSNPs through electrostatic interaction. Complex formation between dsRNA and amino MSNPs was confirmed by a gel retardation assay. Following complex formation, the electrophoretic mobility of dsRNA was markedly reduced, with the dsRNA remaining retained in the wells rather than migrating through the gel, indicating efficient association of dsRNA with the amino-functionalized MSNPs.

Following confirmation of the physicochemical characteristics and dsRNA-loading capacity of the nanoparticles, the uptake and translocation of amino-functionalized MSNPs in *N. benthamiana* leaves and roots were examined using Leica TCS SP5 confocal laser scanning microscope, Leica Microsystems GmbH, Wetzlar Germany Rhodamine B was incorporated into the MSNPs to enable visualization of nanoparticle uptake and localization within plant tissues. As shown in Figure 2C, leaves treated with free Rhodamine B showed a relatively uniform fluorescent signal throughout the treated leaves, indicating absorption and distribution of the dye. However, no detectable fluorescence was observed in adjacent untreated leaves. In contrast, leaves treated with rhodamine B-labeled MSNPs showed fluorescence not only in the treated leaves but also in adjacent leaves, suggesting that the MSNPs were capable of moving from the treated tissue to neighboring leaves through the apoplastic pathway (Figure 2C(i,ii)).

A similar pattern was observed following root application. Free rhodamine B showed relatively uniform fluorescence throughout the roots and leaves, consistent with passive uptake and distribution of the dye. In contrast, plants treated with rhodamine B-labeled MSNPs exhibited localized fluorescence in root and leaves rather than uniform distribution. Distinct fluorescence signals were detected within root and leaves cells following nanoparticle application, demonstrating cellular uptake of the MSNPs (Figure 2C(iii,iv)).

### 2.3. Foliar Applied dsRNA Is Mobile and Translocate from Leaf to Different Parts of Plants

**Figure 3:**
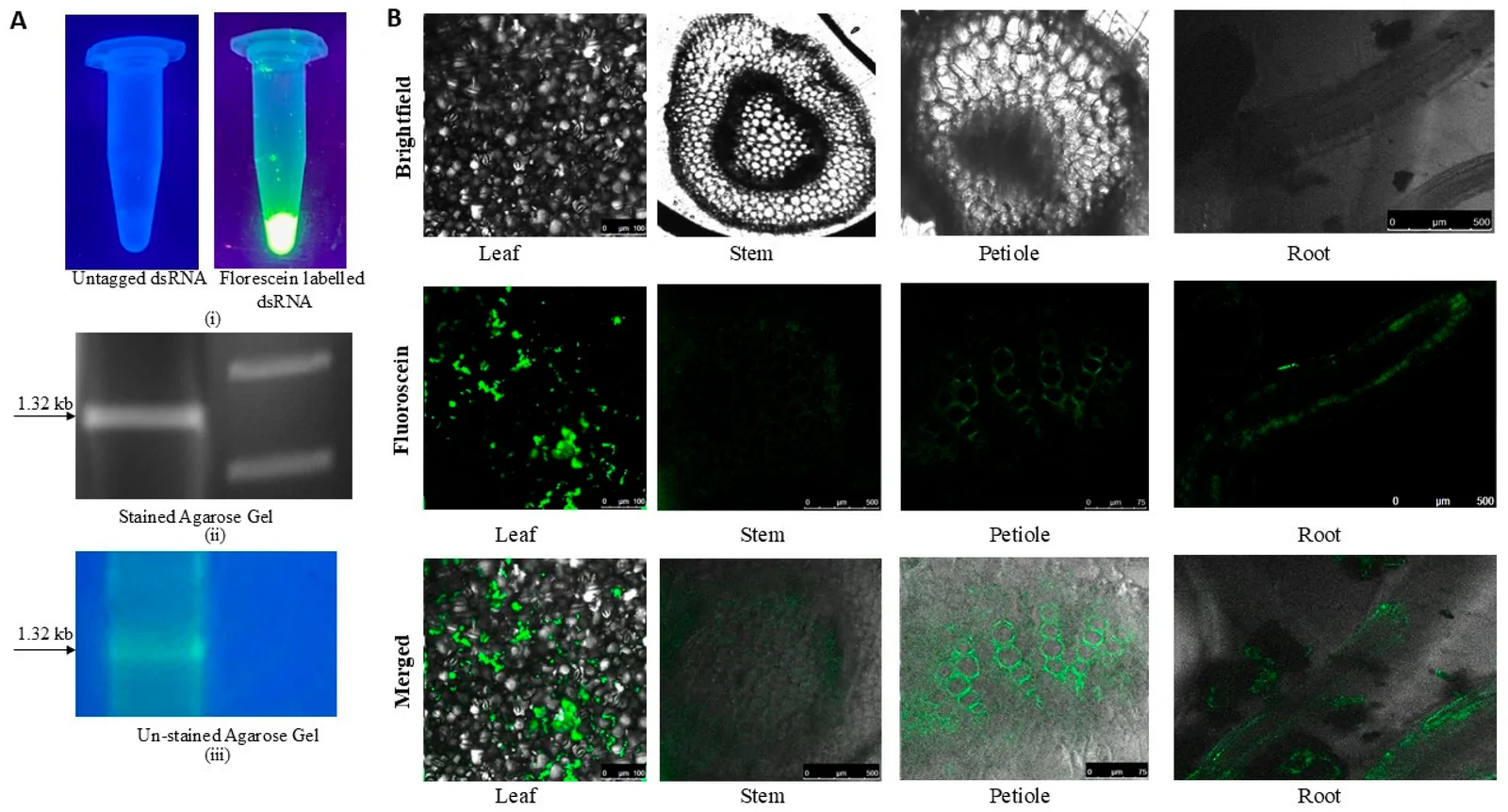
Synthesis and translocation of fluorescein-labeled NSs-specific dsRNA (dsNSs) in *Nicotiana benthamiana*. (**A**) Synthesis and confirmation of fluorescein-labeled dsRNA. (**i**) Fluorescein-labeled dsNSs showing green fluorescence under UV illumination. (**ii**) Confirmation of synthesized dsNSs by agarose gel electrophoresis, along with (**iii**) visualization of its fluorescence in the absence of an agarose gel stain. (**B**) Translocation of fluorescein-labeled dsNSs in different parts of the plant, including leaves, stems, petioles, and roots.

To investigate the translocation of exogenously applied dsRNA within plants, fluorescein-labeled dsRNA was synthesized and its successful labeling was confirmed by agarose gel electrophoresis. Under UV illumination, fluorescein-labeled dsRNA exhibited a distinct bluish-green fluorescence, whereas no fluorescence was observed for the unlabeled dsRNA. Notably, the fluorescein-labeled dsRNA could be visualized directly under UV illumination without additional staining of the agarose gel, further confirming successful incorporation of the fluorescent label (Figure 3A).

**Figure 4:**
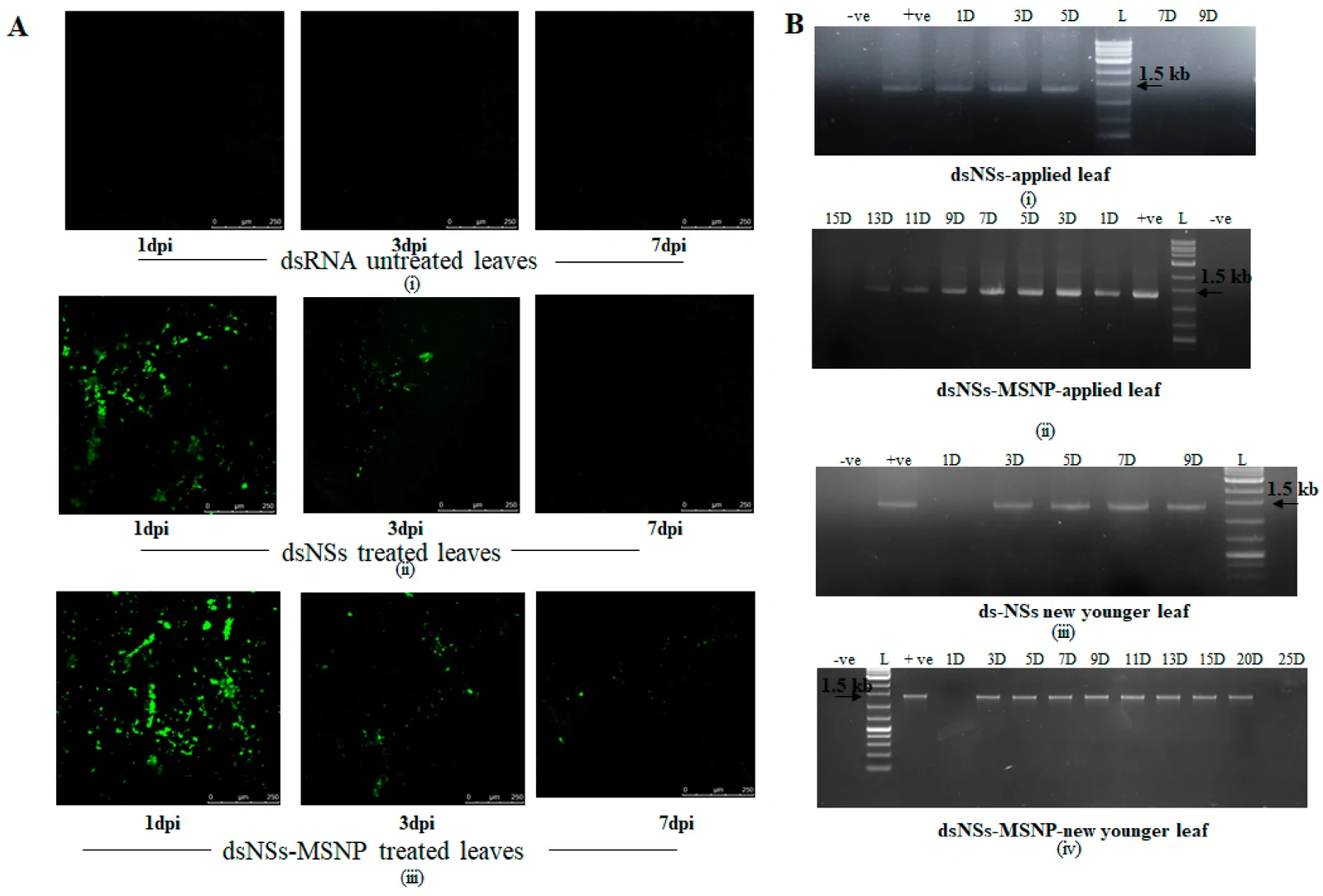
Stability analysis of NSs-specific dsRNA (dsNSs) in *Nicotiana benthamiana* at different time points. (**A**) Confocal laser scanning microscopy (CLSM) analysis of dsNSs and mesoporous silica nanoparticle-conjugated dsNSs (dsNSs-MSNP) in the (**i**) dsRNA untrated leaves, (**ii**) dsNSs treated leaves & (**iii**) dsNSs-MSNP treated leaves at 1, 3, and 7 days post-inoculation (dpi). (**B**) RT-PCR analysis using NSs gene-specific primers to detect dsNSs and dsNSs-MSNP in (**i**,**iii**) treated leaves and (**ii**,**iv**) newly emerged leaves at different time points; 1.2% agarose gel was used; L: DNA marker 1 kb plus DNA ladder (G-Biosciences, USA); D: dpi; −ve: no-template control -; +ve: PCR positive control.

Following foliar application, fluorescence signals corresponding to the labeled dsRNA were detected in the treated leaves as well as in the petiole, stems and roots within 24 h of application (Figure 3B). Because the fluorescein-labeled dsRNA was applied only to the leaves, its detection in these distal tissues demonstrated that exogenously applied dsRNA was capable of translocating from the treated leaf to other parts of the plant. The persistence and distribution of naked dsRNA and MSNP-conjugated dsRNA were subsequently monitored using fluorescence imaging. The fluorescence signal associated with dsNSs in the treated leaves gradually decreased over time, with a reduced signal observed at 3 dpi and no detectable fluorescence at 7 dpi. Similarly, the fluorescence signal from dsNSs–MSNP complexes decreased progressively at 1, 3, and 7 dpi, and no detectable fluorescence was observed beyond 7 dpi (Figure 4A).

To further assess the persistence and movement of dsRNA at the molecular level, RT-PCR was performed using NSs-specific primers. The naked dsNSs was detected in the treated leaves up to 5 dpi (Figure 4B(i)) and in newly emerged newly emerged leaves up to 9 dpi (Figure 4B(iii)). In contrast, dsNSs-MSNP showed prolonged persistence and was detected from 3 dpi onward, remaining detectable in the treated and newly emerged leaves up to 13 (Figure 4B(ii)) and 20 dpi, respectively (Figure 4B(iv)).

### 2.4. dsNSs Conjugated MSNP Protects Nicotiana benthamiana and Cowpea Against WBNV

The protective efficacy of the dsNSs and hpNSs was evaluated in 30-day-old *N. benthamiana* plants containing four to five fully expanded leaves (*n* = 3) and in primary leaves of cowpea cv. Pusa Komal (*n* = 3). Following treatment with the respective RNAi constructs, plants were challenged with WBNV, and disease development and viral accumulation were monitored.

**Figure 5:**
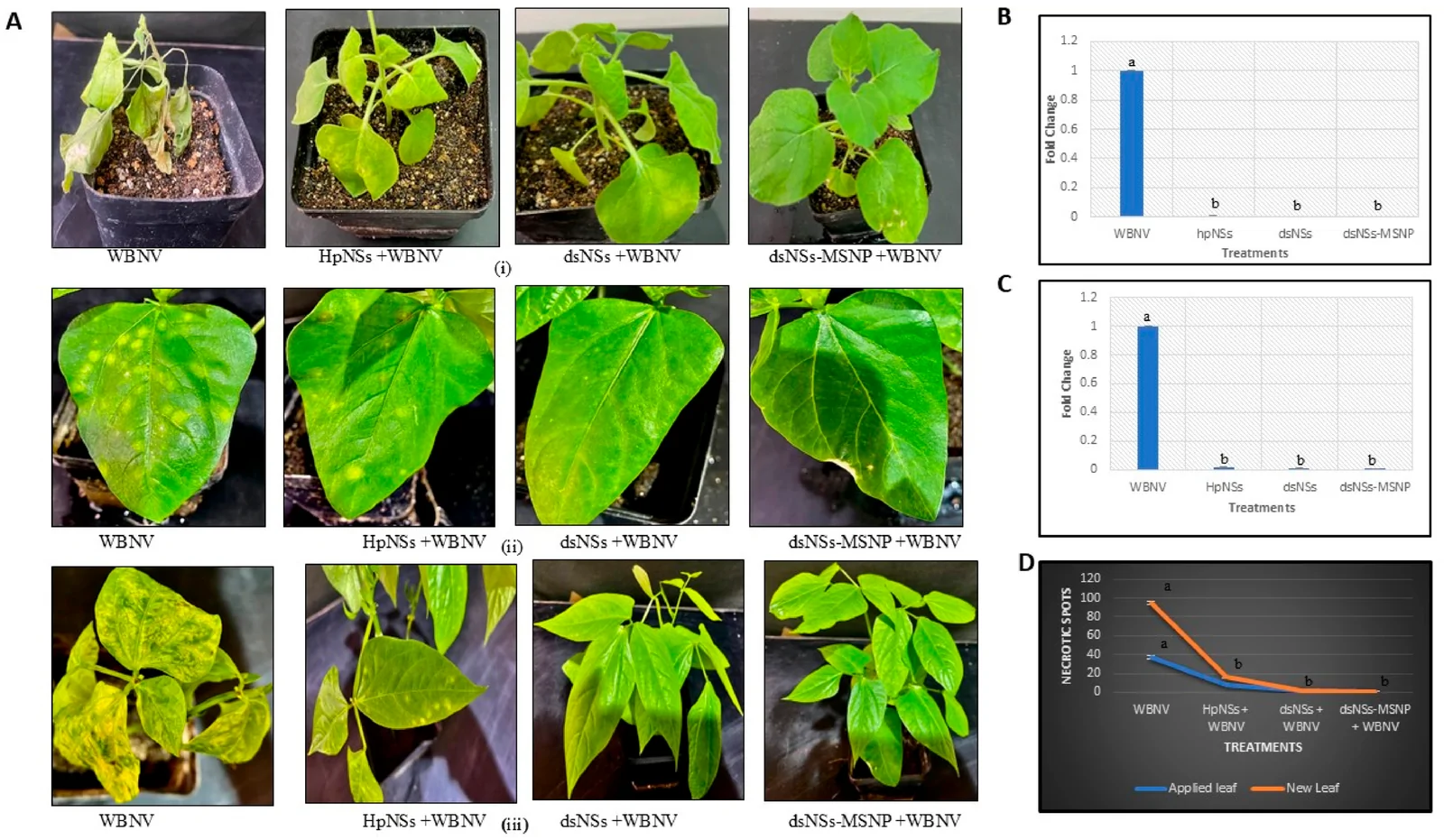
Effect of topical application of NSs hairpin RNA (hpNSs), NSs-specific dsRNA (dsNSs), and mesoporous silica nanoparticle-conjugated dsNSs (dsNSs-MSNP) targeting the *NSs* gene of watermelon bud necrosis virus (WBNV) in *Nicotiana benthamiana* and cowpea challenged with WBNV. (**A**) Symptom development following topical application of hpNSs, dsNSs, and dsNSs-MSNP in WBNV-challenged plants. (**i**) *N. benthamiana* showing reduced symptom development in treated leaves. Cowpea showing reduced symptom development in (**ii**) treated leaves and (**iii**) newly emerged leaves. (**B**,**C**) Quantitative reverse transcription PCR (qRT-PCR) analysis showing a significant reduction (*p* ≤ 0.05) in WBNV RNA accumulation following all three treatments in *N. benthamiana* and cowpea, respectively. (**D**) Necrotic spot development showing a reduction following treatment with hpNSs, dsNSs, and dsNSs-MSNP. Different letters indicate statistically significant differences (*p* ≤ 0.05). *Actin* and glyceraldehyde-3-phosphate dehydrogenase were used as endogenous reference genes in *N. benthamiana* and cowpea, respectively.

Plants treated with hpNSs, dsNSs and dsNSs-MSNP remained nearly symptom-free until 10, 10 and 15 dpi respectively, whereas untreated plants developed chlorotic symptoms from 5 dpi, followed by necrosis, shoot bending, wilting, and plant death. Consistent with the delayed symptom development, hpNSs-, dsNSs- and dsNSs-MSNP-treated plants exhibited a substantial reduction in viral accumulation compared with untreated plants. The relative reduction in viral RNA accumulation was 112-, 303- and 370-fold in *N. benthamiana* (Figure 5A(i),B).

Similarly, cowpea plants treated with with hpNSs, dsNSs and dsNSs-MSNP showed no or very less chlorotic symptom on WBNV inoculation (Figure 5A(ii)). Quantitative analysis of viral accumulation further supported the protective effects of hpNSs, dsNSs and dsNSs-MSNP treatments. Compared with untreated plants, hpNSs treatment resulted in a 76.9-fold reduction in viral RNA accumulation, whereas dsNSs and dsNSs-MSNP treatment resulted in 166 and 208 fold reductions in viral RNA accumulation in *N. benthamiana* and cowpea, respectively (Figure 5C).

In cowpea, severe chlorotic symptoms developed in untreated plants from 6 dpi onward. In contrast, plants treated with hpNSs, dsNSs, or dsNSs-MSNP exhibited substantially reduced disease severity. The percentage reduction in chlorotic spots in the treated leaves was 77.5%, 95.6%, and 98.2% for hpNSs, dsNSs, and dsNSs-MSNP treatments, respectively. In newly emerged leaves, the corresponding reductions were 86.71%, 100%, and 100%, respectively (Figure 5A(iii),D).

## 3. Discussion

WBNV is an important viral constraint of watermelon production in the Indian subcontinent and the occurrence of WBNV has also been reported from several economically important cucurbit crops. Although, RNAi-based approaches have been investigated against several plant viruses, including several orthotospoviruses [21,23,24], the potential of exogenously applied NSs-specific dsRNA for WBNV management has not been investigated in the past. The present study provides the first demonstration of effectiveness of exogenous dsRNA-mediated protection of plant against WBNV.

A critical factor determining the effectiveness of externally applied dsRNA is its ability to enter the plant tissues and move beyond the site of application providing systemic protection against virus disease [25]. In the present study, fluorescein-labeled dsRNA was successfully synthesized and the fluorescence was subsequently observed in the treated leaf, petiole, and stem within 24 h of foliar application. As the dsRNA was applied only to a specific leaf, its detection in distal tissues indicated that the applied RNA was not restricted to the site of application but was capable of moving within the plant system. This observation is consistent with recent evidence demonstrating that foliar-applied dsRNA can enter the vascular system and move to distal aerial and belowground tissues [26]. This study showed that the NSs dsRNA was detected via PCR in newly emerged leaves at 3 dpi and remained detectable up to 9 dpi further supports the systemic movement of externally applied NSs dsRNA.

The movement observed for dsRNA was accompanied by uptake and translocation of amino-functionalized MSNPs. The uptake experiments demonstrated fluorescence from rhodamine-labeled MSNPs in treated leaves as well as adjacent leaves following foliar application, while fluorescence was also detected within root cells and leaves following root application. These observations are consistent with our previous studies demonstrating that mesoporous silica nanoparticles can enter plant and translocate to different parts of plants [27]. Sun et al. demonstrated that 20 nm MSNP penetrate into the roots with both symplastic and apoplastic pathways and then translocate to the conducting tissues of the xylem to the aerial parts of the plants including the stems and leaves [28]. Similarly, Torney et al. demonstrated that functionalized MSNPs can enter plant cells and intact leaves and can serve as carriers for DNA delivery [29]. Foliar-applied MSNP formulations have also been detected in stems, petioles, and roots, demonstrating that silica-based nanocarriers can undergo systemic distribution following application to a single leaf. Our findings therefore support the use of amino-functionalized MSNPs as potential carriers for transporting dsRNA into and within plant tissues.

The ability of MSNPs to facilitate dsRNA delivery is particularly important because the biological activity of naked dsRNA is limited by its susceptibility to degradation and by barriers associated with its uptake into plant cells [25]. In our experiments, naked dsNSs was detected in the treated leaves only up to 5 dpi and in newly emerged leaves up to 9 dpi. In contrast, MSNP-associated dsNSs was detected in the treated leaves up to 13 dpi and in newly emerged leaves up to 20 dpi. Thus, conjugation with MSNPs markedly extended the period during which the dsNSs remained detectable in both treated and newly emerged leaves. Previous work with amino-functionalized MSNPs has demonstrated protection of dsRNA against nuclease degradation and efficient uptake into both leaves and roots of *N. benthamiana*. Importantly, size-dependent uptake of amino MSNPs carrying dsRNA was associated with enhanced antiviral activity against tomato leaf curl New Delhi virus [27,30]. Fluorescence imaging showed a progressive decrease in signal intensity in the treated leaves from 3 dpi onward. No detectable fluorescence signal was observed beyond 7 dpi in leaves treated with naked dsNSs, whereas a weak fluorescence signal was still observed in dsNSs-MSNP-treated leaves at later time points. The difference between fluorescence-based detection and RT-PCR-based detection likely reflects the different detection characteristics of the two approaches. While fluorescence imaging provides a visual indication of the detectable labeled dsRNA-associated signal, RT-PCR targeting the full-length dsNSs region confirmed the presence of full-length dsNSs at later time points. Therefore, the absence of detectable fluorescence does not necessarily indicate complete disappearance of dsRNA. However, the present study did not directly assess the generation or accumulation of dsNSs-derived siRNAs following dsRNA application. Detection of dsNSs-derived siRNAs would provide additional evidence for the processing of the applied dsRNA into RNAi-active molecules and would help clarify the relationship between dsRNA persistence and RNAi-mediated antiviral activity. Therefore, profiling dsNSs-derived siRNAs at different time points following application represents an important scope for future studies.

The effectiveness of the NSs-targeting hairpin construct further demonstrates the suitability of NSs as an RNAi target for WBNV. Plants treated with hpNSs showed markedly delayed symptom development compared with untreated WBNV-infected plants. In *N. benthamiana*, hairpin-treated plants remained symptom-free until 10 dpi. A substantial reduction in viral accumulation was also observed following hairpin treatment. Similar protection was observed in cowpea, where hpNSs substantially reduced disease severity in both treated and newly emerged leaves. These findings indicate that intracellular production of NSs-specific RNAi triggers through the hairpin construct can effectively suppress WBNV multiplication and disease development.

Although naked dsNSs was effective in reducing WBNV accumulation, its efficacy was lower than that observed with the MSNP-associated formulation [31]. The rapid decline in detectable naked dsNSs in the treated tissues is therefore likely to limit the duration over which sufficient RNAi trigger is available. Nevertheless, the detection of naked dsNSs in newly emerged leaves from 3 dpi onward and its association with reduced virus accumulation indicate that transient exposure can generate a systemic antiviral effect. The systemic detection of naked dsRNA is also consistent with recent experimental evidence demonstrating that foliar-applied dsRNA can move through the plant vasculature and reach distal tissues [26,32].

In contrast, MSNP conjugation substantially enhanced the persistence and antiviral activity of dsNSs. Fluorescence imaging showed a progressive reduction in the signal of both naked dsNSs and dsNSs-MSNPs with time, with no detectable fluorescence beyond 7 dpi. This difference between fluorescence and PCR detection is important because the two assays measure different parameters. Fluorescence imaging depends on the persistence and intensity of the fluorescent label, whereas PCR detects the presence of the target NSs sequence and can therefore remain positive even after the fluorescent signal has fallen below the imaging detection threshold. Thus, the PCR results provide stronger evidence for prolonged persistence of the dsRNA sequence in MSNP-treated plants. In *N. benthamiana* and cowpea, dsNSs and dsNSs-MSNP treatment resulted significant reductions in viral load. The greater reduction observed with dsNSs MSNP suggests that extending the residence time and availability of the RNAi trigger can enhance suppression of WBNV replication.

Taken together, our findings demonstrate that topical application of NSs-specific dsRNA can suppress WBNV infection and that the antiviral effect can be substantially improved through nanoparticle-mediated delivery. These findings establish dsNSs-MSNP as a promising non-transgenic platform for WBNV management and provide a foundation for future optimization. Further evaluation under greenhouse and field conditions will be necessary to determine the practical feasibility of this approach for WBNV management.

## 4. Material and Methods

### 4.1. Maintenance of Virus Inoculum and Plants

Samples showing symptoms of WBNV infection were collected from the IARI, New Delhi, field and screened for the presence of the virus. Viral infection was confirmed by PCR using the primer pair BM01-F and BM02-R (Table 1). PCR amplification was performed with an initial denaturation at 94 °C for 5 min, followed by 35 cycles of denaturation at 94 °C for 30 s, annealing at 56 °C for 30 s, and extension at 72 °C for 30 s, with a final extension at 72 °C for 5 min. PCR-positive samples were subsequently used to propagate the virus under controlled conditions in *N. benthamiana* and cowpea plants. The plants and virus inoculum were maintained in a growth chamber at 25 ± 2 °C under a 16 h light/8 h dark photoperiod.

### 4.2. Generation of Plasmid Constructs

The full-length *NSs* gene of WBNV was amplified by RT-PCR using the cDNA synthesized from total RNA extracted from a WBNV-infected culture, using primer set BM196-F and BM197-R (Table 1). The amplified NSs fragment was initially cloned into the pGEM-T Easy vector and sequenced and subsequently subcloned into the L4440 vector using the HindIII and SalI restriction sites. For construction of the hairpin RNA interference (hpRNAi) construct, the *NSs* gene was amplified using primer set BM1299-F and BM1300-R containing a BsaI restriction site followed by a five-nucleotide fusion sequence required for assembly with the inverted repeat region of the pRNAiGG vector (Table 1). The amplified products were used for Golden Gate assembly to generate the pRNAiGG-NSs construct following the protocol described by [33]. The Golden Gate reaction contained 200 ng of PCR product, 200 ng of pRNAiGG vector, 0.5 µL of T4 DNA ligase (5 U/µL; Thermo Fisher Scientific, Waltham, MA, USA), 1 µL of 10× T4 DNA ligase buffer, and 0.5 µL of BsaI enzyme (10 U/µL; Thermo Fisher Scientific, Waltham, MA, USA). The reaction was performed in a thermocycler at 37 °C for 5 min and 16 °C for 10 min, with these steps repeated for 10 cycles, followed by incubation at 50 °C for 5 min and 80 °C for 5 min. The assembled products were used to transform *E. coli* DH5α, and the putative positive clones were screened by colony PCR using gene-specific forward and reverse primers. Plasmid PCR was additionally performed using the P21-RNAiGG-F and P22-RNAiGG-R primers in combination with the respective gene-specific reverse primers BM 197R to verify the correct orientation and assembly of the NSs fragment within the vector (Table 1). The confirmed hpNSs constructs were subsequently used to transform *Agrobacterium tumefaciens* strain GV3101.

### 4.3. Purification of dsRNA

The *E. coli* HT115 cells was transformed with the confirmed NSs-L4440 recombinant plasmid into for dsRNA production. A positive HT115 colony was picked and grown overnight, which served as the primary culture. In the following day, the secondary bacterial culture was prepared from the overnight culture, grown to an optical density (OD600) of 1.0 and induced with 0.4 mM IPTG for 4 h to facilitate dsRNA expression. The dsNSs was subsequently purified from 100 mL of the IPTG-induced bacterial culture following the method described by Gupta et al. [24]. Briefly, the bacterial cells were harvested by centrifugation, and the resulting pellet was resuspended in 1× phosphate-buffered saline (PBS) containing 70% (*v*/*v*) ethanol and incubated at 4 °C for 30 min. The cells were then resuspended in 2 mL of 150 mM NaCl, followed by precipitation of the extracted dsRNA with absolute ethanol.

### 4.4. Synthesis of MSNP, Size and Charge Characterization and dsRNA Conjugation

MSNPs were synthesized using tetraethyl orthosilicate (TEOS) as the silica precursor and triethylamine (TEA) as the base catalyst. The nanoparticle synthesis, size estimation, surface functionalization of MSNPs, and subsequent conjugation with dsRNA were performed following the protocol described in our previous study, Sangwan et al. [27]. The size estimation of the synthesized MSNP was done by transmission electron microscopy, and zeta potential measurement for amine-functionalized MSNP was conducted on a Malvern Zetasizer Nano-series (Malvern Panalytical, Malvern, UK) as described in Sangwan et al. [27].

### 4.5. Movement of MSNP in N. benthamiana Plants

To investigate the movement and uptake of MSNP in plants, the nanoparticles were fluorescently labeled with Rhodamine B. Briefly, 2 mg of Rhodamine B was dispersed in 50 mL of deionized water under continuous stirring at 25 °C. After 15 min, 0.05 g synthesized MSNPs was added to the solution, and the mixture was further stirred for 50 min. The nanoparticles were subsequently recovered by centrifugation and washed thoroughly with deionized water to remove unbound dye.

For translocation study, 0.25 mg of Rhodamine B-labeled MSNPs was dispersed in deionized water and applied to the leaf surface. As a control, 100 µL of Rhodamine B solution (2 mg/50 mL) was applied separately to the leaf surface. To assess root uptake, the roots of one-month-old plants were submerged in 1.5 mL microcentrifuge tubes containing 0.5 mg of Rhodamine B-labeled MSNPs dispersed in deionized water. Following treatment, both leaf- and root-treated plants were kept for 24 h and subsequently washed thoroughly with deionized water to remove surface-associated nanoparticles. Fluorescence associated with MSNP uptake and movement in leaf and root tissues was then monitored using a Leica TCS SP5 confocal laser scanning microscope, Leica Microsystems GmbH, Wetzlar, Germany.

### 4.6. Fluorescein Labeling of dsRNA

To study the movement of dsNSs within the plant system, fluorescein labeled dsRNA corresponding to the *NSs* gene of WBNV was synthesized. Primers were designed to amplify the *NSs* gene, with the T7 promoter sequence (5′-TAATACGACTCACTATAG-3′) incorporated at the 5′ end of both the forward and reverse primers. The NSs fragment was amplified using 100 ng of the NSs recombinant plasmid as template in a reaction mixture containing 1× DreamTaq buffer, 1 U of DreamTaq DNA polymerase, 10 mM dNTPs, and gene-specific forward and reverse primers with T7 promoters BM1343-F and BM1344-R (Table 1) [34]. PCR amplification was performed with an initial denaturation at 95 °C for 3 min, followed by 35 cycles of denaturation at 95 °C for 30 s, annealing at 52 °C for 30 s, and extension at 72 °C for 30 s. The amplified product was analyzed by agarose gel electrophoresis and subsequently purified.

Fluorescein-labeled dsNSs was synthesized using the purified PCR product as the template. A 1 µg quantity of PCR product was combined with 2 µL fluorescein RNA-labeling mix (Roche, cat No. 11685619910), 1× transcription buffer, 20 unit of T7 RNA polymerase (Thermo Fisher Scientific, Waltham, MA, USA), and nuclease-free water to obtain a final reaction volume of 20 µL. The reaction mixture was gently mixed by pipetting and briefly centrifuged before incubation at 37 °C for 16 h. The synthesized fluorescently labeled dsNSs was analyzed by agarose gel electrophoresis and subsequently purified using a gel elution kit. Successful fluorescent labeling was further verified by electrophoresis of the synthesized dsNSs on an agarose gel without staining, allowing for direct visualization of the fluorescent signal. To confirm the identity of the synthesized nucleic acid as dsRNA, the sample was subjected to nuclease free DNase I and RNase H digestion under low- and high-salt conditions, as described by Gupta et al. [24].

### 4.7. Movement of dsRNA in Plant System

The movement and distribution of exogenously applied, fluorescein-labeled dsRNA within the plant system were assessed in *N. benthamiana* plants; 10 µg of labeled dsNSs was suspended in 0.01% Celite^®^ 545 and applied to the leaves of 2–3-week-old *N. benthamiana* plants. Following treatment, dsRNA adsorption and uptake by the leaf tissue were assessed by monitoring the fluorescent signal using a Leica TCS SP5 confocal laser scanning microscope at an excitation/emission wavelength range of 490–520 nm, with a 100 µm pinhole and EMP filter. The treated leaves were thoroughly washed; 4 mm leaf discs, thin sections of the stem and petiole, and small root segments were excised; and the movement and distribution of the labeled dsNSs to different plant tissues were subsequently monitored using the Leica TCS SP5 confocal laser scanning microscope.

### 4.8. In Planta Stability Assessment of dsNSs and dsNSs-MSNP

The degradation patterns of dsNSs and dsNSs-MSNP were assessed by RT-PCR using primers targeting the full-length *NSs* gene with primer set BM 196 and BM 197. Samples from the treated leaves were collected at one-day intervals from 1 to 15 dpi, while samples from newly emerged leaves were collected up to 25 dpi for assessment by RT-PCR. dsNSs and dsNSs-MSNP were applied to the plants at concentrations similar to those described in Section 4.9.

### 4.9. Topical Application of hpNSs, dsNSs and dsNSs-MSNP in N. benthamiana and Cowpea

The efficacy of dsNSs against WBNV was assessed in *N. benthamiana* and cowpea. Each treatment consisted of three biological replicates (three plants), and the experiment was independently repeated three times. dsNSs was dissolved in deionized water with 0.01% Celite^®^ 545 at a total concentration of 10 µg and were applied by gently rubbing onto the adaxial surface of leaves of 30-day-old *N. benthamiana* and 6-day-old cowpea plants at two leaves stage. Further, for application of dsNSs-MSNP, approximately 10 µg/mL of dsNSs conjugated with MSNP at a weight ratio of approximately 1:6.67 as described in our previous study Sangwan et al. [27]. dsNSs-MSNPs were applied on the adaxial leaf surface of *N. benthamiana* and cowpea by gentle rubbing. For both dsNSs and dsNSs-MSNP treatments, simultaneous application of the treatments and inoculation with WBNV was performed in *N. benthamiana* and cowpea plants. The PCR positive *Agrobacterium tumefaciens* GV301 colony harboring hpNSs against WBNV were applied onto the bottom portion near the petiole of *N. benthamiana* and cowpea leaves according to the protocol described in Sarkar et al. [35], followed by the challenge inoculation of WBNV on the same leaf two days post-hpRNAi construct inoculation.

### 4.10. Challenge Inoculation with Watermelon Bud Necrosis Virus

The challenge inoculation of dsNSs and hpNSs treated plant with WBNV was performed as describe by Rai et al. [36]. The WBNV-inoculated *N. benthamiana* leaves showing initiation of mottling and bending symptoms were used to prepare inoculum in the extraction buffer at a ratio of 1:6 (1.0 g of leaves in 6 mL of extraction buffer) with a pre-chilled mortar and pestle. The extraction buffer was composed of 0.1 M sodium phosphate buffer, pH 7.0, 0.002% beta-mercaptoethanol. The test plants were pre-dusted lightly with Celite^®^ 545 powder and then 100 μL of the prepared WBNV inoculum was applied gently on the surface of each leaf (3 leaves per plant). The inoculated plants were sprayed with distilled water and maintained in a controlled environment room for symptom expression.

### 4.11. Quantification of Viral Load in dsRNA Treated Plants

For the detection of WBNV viral load, real-time PCR was employed. Initially, cDNA was synthesized from 1.0 μg of isolated samples using random hexamers following the instructions provided in the Revert Aid First Strand cDNA Synthesis Kit (Thermo Fisher Scientific, Waltham, MA, USA). Subsequently, quantitative PCR (qPCR) was conducted using 1.0 μL of cDNA, along with gene specific primers (BM1345-F and BM 02-R) designed for amplification of a partial fragment of the *NP* gene of WBNV as a viral target gene. The *actin* gene primer set BM849-F and BM850-R and glyceraldehyde-3-phosphate dehydrogenase (GAPDH) primer set BM1261-F and BM1262-R was utilized as an endogenous gene for normalization purposes in *N. benthamiana* and cowpea, respectively. The relative reduction of viral load in both treated and untreated plants was calculated utilizing the double Ct method, as described by Livak and Schmittgen [37], with the viral load in untreated plants set as 1 for comparison.

### 4.12. Statistical Analysis of Data

To determine the statistical significance of plants treated with hpNSs, dsNSs, and dsNSs-MSNP from the control plants (only virus-inoculated), Student’s *t*-test was carried out.

## Figures and Tables

**Table 1:** List of primers used in this study.

Name	Sequence	Annealing Temp	Use
BM01-F	TTGGATCCATGTCTACCGTTAAGCAACT	56.0	To amplify *NP* gene of WBNV
BM02-R	AAGAGCTCTTACACTTCCAAAGAAGTGC		
BM196-F	ATGTCTAACGAAAAGAGTGTT	55.0	To amplify *NSs* gene of WBNV
BM197-R	TCAGTTAGCGAATCACAACAAAAT		
BM1299-F	TTACCAGGTCTCAGGAGATGTCTAACGAAAAGAGTGTT	60.0	To amplify *NSs* gene for cloning in RNAiGG vector
BM1300-R	AATACCAGGTCTCATCGTATCAGTTAGCAATCACAACAAA		
P21-RNAIGG-F	ACCATTTACGAACGATAGCC	54.0	To confirm the inverted arm of hairpin construct
P22-RNAIGG-R	GTAAAACGACGGCCAGTG		
BM849-F	GGTATTGTGTTGGACTCGGG	60.0	Partial actin gene used as a reference gene
BM850-R	GCTGTGGTAGTGGATGAGTAAC		
BM1261-F	ATCAGCCAAGGACTGGAGAG	58.0	Partial GAPDH gene used as a reference gene
BM1262-R	ACGGAATGCCATACCAGTCA		
BM1343 * F	gaattaatacgactcactatagggagaATGTCTAACGAAAAGAGTGTT	58.0	NSs gene with T7 Promoter
BM1344 * R	gaattaatacgactcactatagggagaATCAGTTAGCAATCACAACAAA		
BM1345-F	GCCTTGGATAACATGTTCAAA	60.0	NP gene real-time primer

* lower letter indicates the T7 binding site.

## Data Availability

All relevant data is provided in figures and table in the manuscript.

## Supplementary Materials

The following supporting information can be downloaded at: https://www.mdpi.com/article/10.3390/plants15203237/s1, Figure S1: Zeta potential of amine-functionalized MSNPs measured using a Malvern Zetasizer Nano Series. NP indicates unfunctionalized MSNP; NP* indicates amine-functionalized MSNPs.
